# Synthesis of a novel Ce(iii)/melamine coordination polymer and its application for corrosion protection of AA2024 in NaCl solution

**DOI:** 10.1039/d0ra08587a

**Published:** 2021-02-03

**Authors:** Mahmoud Zorainy, Daria C. Boffito, Mohamed Gobara, Ahmad Baraka, Ibrahim Naeem, Hesham Tantawy

**Affiliations:** Chemical Engineering Department, Military Technical College Cairo Egypt h.tantawy@mtc.edu.eg ramzy.hrt@gmail.com; Chemical Engineering Department, Polytechnique Montréal Montreal Canada

## Abstract

We present the synthesis of a new cerium(iii)–melamine coordination polymer (CMCP) by a mixed-solvothermal method and its characterization. Characterization techniques included Raman, Fourier Transformation Infra-Red (FTIR), X-Ray Diffraction (XRD) and Scanning Electron Microscope (SEM), in which the change in the electronic environment and the crystallinity were tracked. The characterization results confirm the coordination of cerium(iii) with melamine through –NH_2_ groups, instead of the N atoms of the triazine ring, for which we propose a mechanism of interaction. In addition, Biovia Materials Studio package was applied to determine and investigate the molecular structure of the CMCP. All simulations were done using COMPASS force-field theory and atom-based method for summation of electrostatic and van de Waals forces. The application of the CMCP for the corrosion inhibition of AA2024 in 3.5% NaCl solution was tested using the potentiodynamic polarization and electrochemical impedance spectroscopy (EIS) techniques. The results point out that the presence of cerium as cerium(iii) in the CMCP structure plays the fundamental role of inhibition, whereby the inhibition mechanism occurs by cathodic oxidation of Ce(iii) to Ce(iv) and cyclic reduction of Ce(iv) to Ce(iii) by melamine part of CMCP.

## Abstract 2

We present the synthesis of a new cerium(iii)–melamine coordination polymer (CMCP) by a mixed-solvothermal method and its characterization.

## Graphical abstract

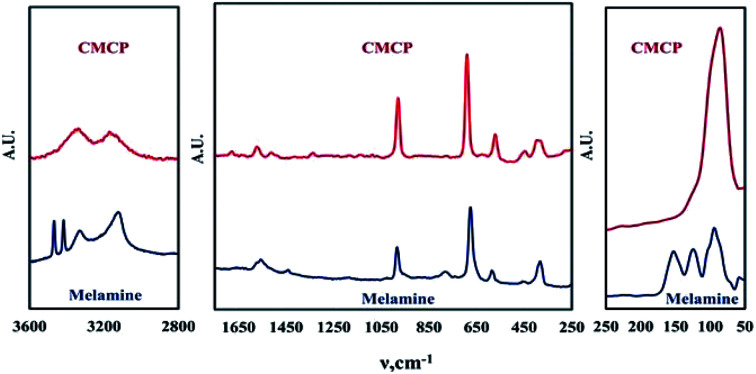

## 1. Introduction

Corrosion inhibition crosscuts several industrial applications, including metals and alloys for pipelines, plant elements and other metallic assets, concrete for the construction industry, heritage artifacts,… *etc.* Aluminium has been widely used in numerous engineering applications due to its many advantages such as recycling, low density, wide range of alloys and ease of surface treatments.^1^ AA2*xxx* is an aluminium alloy series with copper (≈3.8–4.9%) main alloying element. AA2024 is widely used in aircraft applications due to a combination of high strength to weight ratio, high fatigue resistance and toughness.^2^ However, this alloy does not have suitable corrosion resistance in atmospheric conditions; consequently, it is used with cladding and/or coating which usually comprises an inhibitor. Using inhibitors is a cost effective technique during operation and maintenance where using economical materials with inhibitor is much cheaper than using an expensive corrosion resistant material without inhibitor.^3^

Generally, metal corrosion is inhibited, reduced or controlled by adsorption-blocking and/or electrochemical action.^4^ Inhibitors working *via* the adsorption-blocking mechanism form a protective layer by physisorption at the metal/electrolyte interphase or as an insoluble complex barrier constructed by chemisorption, which prevents direct contact between the metal surface and the corrosive medium. Most of these inhibitors are organic materials designed to comprise aromaticity, heteroatoms such as nitrogen, oxygen and/or sulfur and/or conjugation. Indeed, the interaction is most favored with the metal when the inhibitor has a planar conjugated structure with a high π electron density.^5^

Chromate inhibitor/treatments had been effectively used to control the corrosion of AA2024 for many decades. Although, the prices of chromate treatment are cheap with respect to other inhibitors, there is a pressing demand to phase it out due to its carcinogenic effect. This adverse impact governs its concentration limits and finally it would be banned in many countries. Recently, researchers have investigated to replace chromate inhibitors with many eco-friendly inhibitors. Rare earth elements^6–9^ and coordination polymers (CPs) are considered as a suitable replacement candidate. The bonding of rare earth element to an organic matrix would confer the material the advantages of both organic and inorganic inhibitor. Although, the high prices of rear earth elements due to the high demands of other technology, cerium would have suitable stable price due to the fact that its applications in other technologies are well established.^10^

Recently, coordination polymers have been considered for corrosion inhibition, whereby the adsorption mechanism plays the leading role. For example, Etaiw *et al.* proposed Ag(CN)_4_ (quinoxaline)_2_ to inhibit the corrosion of carbon steel in 1 M HCl and found that nitrogen heteroatoms are responsible for the strong adsorption of the material to the metal surface.^11^ The same authors repeated the same type of experiments with Ag(quinoxaline)(4-aminobenzoic acid) as an inhibitor and found that inhibition is ascribed to adsorption as well.^12^

On the other hand, Massoud *et al.* suggested that the inhibition by CPs acts through both anodic and cathodic processes, with a dominant anodic inhibitory effect, when they investigated the use of two silver pyrazine MOFs, [Ag_2_(ampyz)(NO_3_)_2_]_*n*_ and [Ag(2,3-pyzdic)(NO_3_)]_*n*_ as corrosion inhibitors for mild steel in acidic medium.^13^

However, the literature data concerning the use of coordinated polymers (CPs) and metal–organic frameworks (MOFs) for corrosion inhibition are relatively scarce, and they all lead to the same conclusion that inhibition occurs by an adsorption-blocking mechanism.^14–16^ Indeed, CPs and MOFs include a countless number of different compounds where the organic linker confers them different functional groups, electron donor heteroatoms and p-orbital characters.^4^ On the other hand, and to the best of our knowledge, the corrosion inhibition by CPs or MOFs through electrochemical action has not been reported yet.

The use of a CP or a MOF for inhibition through electrochemical action requires a moiety in the structure that is suitable for a redox reaction, *i.e.*, a moiety that undergoes oxidation/reduction must be present with suitable potential with respect to the metal it protects. Cerium is well known for being used as a corrosion inhibitor.^17–19^ In a corrosive media, Ce(iii) plays the crucial role in the protection mechanism, whereby it precipitates on the cathodic sites as insoluble oxides and/or hydroxide hindering the reduction reactions.^18,20^ Therefore, we speculated that bonding Ce(iii) to melamine as a ligand could form an effective material for metal corrosion inhibition.

Melamine (2,4,6-triamino-*s*-triazine) is considered as a poor ligand as it has a strong network structure due to extensive hydrogen bonding throughout the material, which causes very low solubility in common solvents, except for hot water. Hence literature data on the coordination of neat melamine is poor and mostly concerns its coordination with Ag metal to either purify water or perform ion exchange in aqueous media.^21–23^ Differently, there are many examples concerning the coordination chemistry of either functionalized melamine (hexamethylolmelamine,^24^ Schiff base networks^25^), or its coordination with other organic monomers such as succinic and adipic acid,^26^ uric acid.^27^ However, pure melamine/metal coordination should occur under proper synthetic conditions involving, for instance, an acidic environment.

In this study, we aimed at coordinating Ce(iii) in the form of CP, whereby melamine was used as the coordinating ligand. This work is original for several reasons: it presents the synthesis of this novel CP, cerium/melamine coordination polymer (CMCP) and its characterization using FTIR, Raman, CHNO/S elemental analysis, XRD, and SEM-EDX. Also, it discusses the potential use of CMCP in the corrosion protection of AA2024 in aerated 3.5% NaCl solution. The electrochemical inhibition was studied through two techniques: potentiodynamic polarization (PDP), and electrochemical impedance spectroscopy (EIS). The results of corrosion inhibition by CMCP are compared with those of blank, melamine, and cerium(iii) inhibitors. In addition, SEM/EDX mapping was applied to give a better understanding of the inhibition behavior. This work presents the capability of corrosion inhibition by CPs as a promising valuable application in addition to other vital applications such as catalysis, gas storage, ion exchange, optics, and drug delivery.^28^

## 2. Materials and methods

### 2.1 Synthesis of cerium/melamine coordination polymer (CMCP)

Cerium(iii) sulfate (0.142 g) was dissolved in deionized water (5 ml), and melamine (0.126 g) solubilization was aided by an over-the-counter microwave furnace in acetic acid (15 ml, closed vial). The Ce(iii)/water solution was added to the melamine/acetic acid solution, and the vial was tightly closed and put in a pre-heated oven (70 °C) for a mixed-solvothermal reaction. After about 30 minutes, the white CMCP solid precipitate settled at the bottom of the vial. The precipitate was separated from the reaction solution and was thoroughly washed several times alternating acetic acid and water, and then left to dry at ambient conditions. After that, it was stored in a clean vial until characterization and the corrosion inhibition experiments.

### 2.2 CMCP characterizations

Dry amounts of CMCP, cerium(iii) sulfate Ce_2_(SO_4_)_3_, and melamine were characterized by Raman spectroscopy in the 400–4000 cm^−1^ range using a dispersive Raman microscope (Model Senterra, Bruker, Germany, at laser wavelength 532 nm). In addition, dry amounts of CMCP and its precursors, Ce_2_(SO_4_)_3_ and melamine, were thoroughly mixed separately with KBr and then were pressed to form transparent discs. The FTIR spectra of these discs were recorded in the 400–4000 cm^−1^ range with an automatic signal gain that collected 500 scans at a 4 cm^−1^ resolution with a JASCO Model 4100 spectrometer (Japan). The background spectrum was recorded from the clean empty cell at ambient temperature and was taken into consideration during the analyses. To reveal the crystalline nature of CMCP, powder XRD patterns of CMCP and melamine were recorded in the 2*θ* range from 20° to 60° with a CuKα radiation at 40 kV and 30 mA at a scanning speed of 4 degree per min with a sampling angle interval of 0.04 degree using an X-ray diffractometer (XRD, Shimadzu XD-l).

The surface morphology of the CMCP powder sample was investigated using a scanning electron microscope (SEM, Carl Zeiss EVO-10) equipped with an energy-dispersive X-ray spectroscopy (EDX) analyzer. The semi-quantitative elemental analysis^29,30^ for the prepared CMCP crystals was conducted using EDX.

### 2.3 Aluminum alloy specimen preparation

A sheet of aluminium alloy AA2024 [composition, as determined by wt%: Cu (3.8–4.9), Mg (1.2–1.8), Si (0.50), Fe (0.50), Mn (0.3–0.9), Cr (0.10), Zn (0.25), Ti (0.15), and Al (balance)] was obtained from Q-panel™. The sheet was pressed-cut into coupons where each had the dimensions 3.0 × 2.5 × 0.1 cm^3^. Coupons were degreased and cleaned using ethanol, followed by rinsing with deionized water before drying for about an hour at room temperature. After dryness, the coupons were stored in a desiccator until the use in corrosion experiments. In this study, saline corrosive electrolyte (3.5% aqueous NaCl, Sigma Aldrich) is used, which is the typical salt concentration in sea water. Many researchers have widely used such electrolyte to evaluate the corrosion resistance of aluminium alloys. This aggressive Cl^−^ containing electrolyte is able to damage the passive aluminium oxide layer and significantly cause a localized corrosion attack. In addition, this solution is used as the corrosive electrolyte for AA2*xxx* series in many corrosion evaluation standards such as ASTM G44 and G47.

### 2.4 Electrochemical characterization studies

A potentiostat/galvanostat with three-electrode type cell was the setup to perform the electrochemical corrosion experiments with a Gamry™, model reference 600. For all the experiments, the ratio of corrosive solution volume to sample surface area was adjusted to 50 ml cm^−2^.

For each experiment, a freshly prepared AA2024 coupon constituted the working electrode, saturated calomel was the reference electrode, and platinum the auxiliary electrode. The measurements were conducted according to ASTM standard G59 where the potentiodynamic polarization experiments were performed after immersion of the working electrode for at least 30 minutes in the corrosive solution. The potentiodynamic polarization curves were obtained in the potential range of −250 to +250 mV with respect to open-circuit potential (OCP) at a scan rate of 0.5 mV s^−1^. The inhibition efficiency (IE) was calculated using the following relation:^31^1
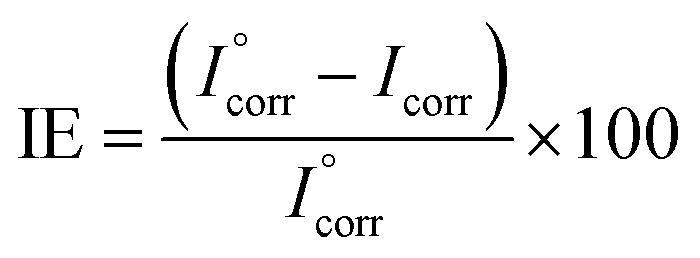
where: 
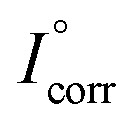
 is the corrosion current density of blank sample and *I*_corr_ is the corrosion current density of inhibited sample.

The polarization resistance (*R*_p_) was also determined to evaluate the corrosion resistance of AA2024 in presence and absence of inhibitor. *R*_p_ was calculated from both anodic and cathodic slopes (*β*_a_ and *β*_c_) of Tafel extrapolation curve according to the following equation:^32–34^2
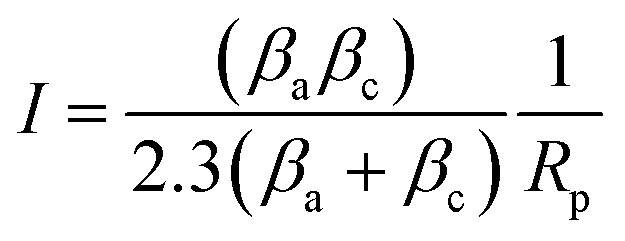


Moreover, the corresponding protection efficiency (PE%) was calculated according to the following equation:3
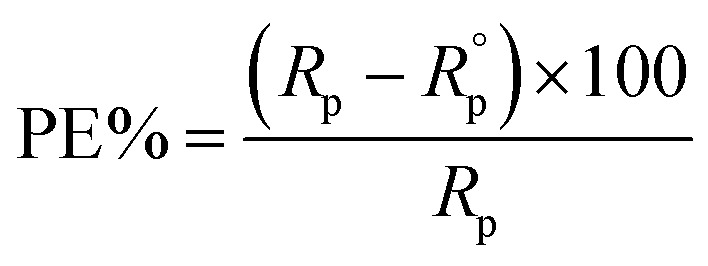
where: *R*_p_ and 
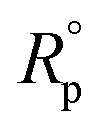
 are the polarization resistances of metal substrate in the electrolyte with and without the additives respectively.

The EIS tests were performed at room temperature by a three-electrode assembly. A saturated calomel electrode was used as a reference electrode, platinum as a counter electrode, and freshly prepared AA2024 was the working electrode. The EIS tests were performed using a Gamry reference 600 instruments in a frequency range of 10^5^ to 10^−2^ Hz with an amplitude of 10 mV peak-to-peak, using AC signal at OCP. The inhibition efficiency (*η*) was calculated using the following relation:^35^4
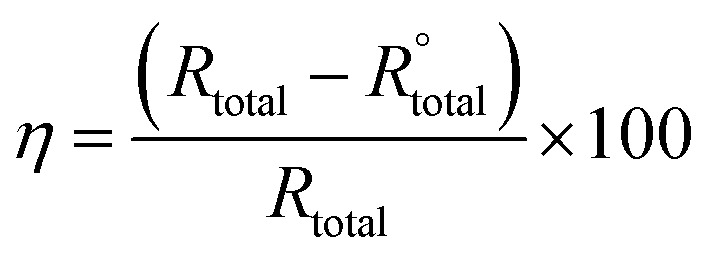
where 
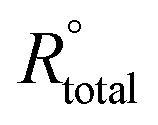
 and *R*_total_ (Ω cm^2^) are the total real resistance in the absence and the presence of inhibitor, respectively.

Fitting the EIS experimental data to equivalent electrical circuits was conducted by applying a nonlinear least square fitting technique using Gamry™ Echem Analyst software version 5.68. The quality of fitting was accepted when the goodness of fitting was not more than 1 × 10^−3^.

## 3. Results and discussion

### 3.1 CMCP characterization

In a first exploratory step, Raman and FTIR spectroscopies identified the structure of the samples based on the electronic environment of the precursors and products. In a second step, XRD and then SEM-EDX inspected the coordination within CMCP and its morphology.

**Fig. 1:**
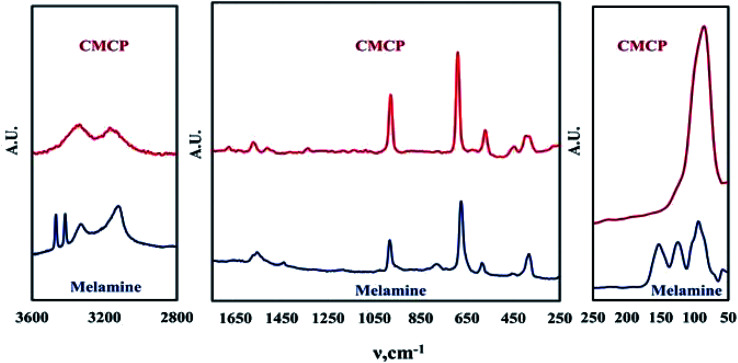
Raman spectra of melamine and CMCP.

The Raman spectrum (Fig. 1) can be divided into three regions. The first region is the low wavenumber region (below 300 cm^−1^), which corresponds to lattice vibrations and is considered key in the case of crystalline materials.^36^ Hence, the presence of peaks in this region for melamine and CMCP indicates that these materials are crystalline. The disappearance and/or change of lattice vibration peaks upon the formation of CMCP from melamine indicates that crystal is changed by any means.^37^ From the figure, the main lattice vibration bands for melamine are present at 57, 93, 123, and 153 cm^−1^.^38–40^ Once CMCP is formed, on the other hand, only one very strong sharp peak appears at 87 cm^−1^. Since lattice vibrations are unique for a given crystalline substance, this peak can be sought as identification of the formation of CMCP.^37^ In general, the observed dramatic change in the lattice vibrations region of the product, CMCP, with respect to that of melamine, indicates that we obtained a crystalline CMCP. In addition, the lack of lattice vibration peaks of melamine in the CMCP spectrum would indicate complete conversion and a pure product.

The second region is the fingerprint region, which lies in the 400–1800 cm^−1^ range.^41,42^ Typical Raman peaks of melamine appears at 380, 582, 676, 780 and 984 cm^−1^.^38,40,42,43^ The most intense one at 676 cm^−1^ is assigned to the ring breathing II mode and involves in-plane deformation of the triazine ring.^44,45^ The second intense peak at 984 cm^−1^ arises from the ring breathing mode I of the triazine ring.^44^ Peaks appearing at 380, 582, and 780 cm^−1^ correspond to the triazine ring as follows: quadrant out-of-plane, ring bending, and out of plane C–N bend, respectively.^46,47^ The weak peak at 1438 cm^−1^ and the broad weak peak around 1560 cm^−1^ belong to ring vibrations and side-chain C–N stretching, respectively.^38,46,48^

Regarding the Raman spectrum of CMCP, it is evident that the melamine fingerprint peaks are collectively still present with some shifts. Furthermore, in this region, these peaks became more intense relative to those of melamine. This indicates that the triazine ring kept its aromatic structure and became even more ordered in the CMCP crystal.^49^ Moreover, the emergence of a new weak peak in the CMCP spectrum at 1678 cm^−1^ may be attributed to a new mode of NH_2_ bending upon complexing.^42,46,50^ The previous discussion leads to the conclusion of coordination of cerium with the amino groups, not with *N*-triazine and the observed shifts in peaks' positions of CMCP to those of melamine may occur merely because of the coordination of NH_2_-melamine molecules with cerium, which changes the electronic environment of the triazine ring. Besides, the observed strong blue-shift of the peak at 675 cm^−1^ (ring bending deformations of melamine) to 690 cm^−1^ (CMCP) confirms the presence of successive Ce(iii)–melamine molecule bonding as the coordination weakens the original triazine ring bond strength.^51^

The last region comprises mainly the N–H vibrations. The melamine spectrum shows four typical peaks: sharp twin peaks at 3468 and 3417 cm^−1^, with two other peaks at 3328 and 3210 cm^−1^. These peaks are attributed to the asymmetric and symmetric N–H stretching.^46,47^ The splitting of the N–H vibrations may be caused by the strong hydrogen bonding between the melamine molecules.^38^ On the other hand, CMCP has only two broad peaks at 3337 and 3165 cm^−1^, and this could be attributed to the distortion of N–H groups of melamine molecules in the CMCP crystal. There are probably no conditions for the strong coupling giving NH bands caused by intermolecular hydrogen bonds N–H⋯N type, like in the crystal of melamine alone.^46^ The significant change in this part of the spectrum of the product confirms the difference of orientation of melamine molecules in the product rather than of the pure melamine.

The Raman spectrum also confirms that CMCP does not contain Ce-oxides, as no corresponding peaks are observed. In addition, there are no peaks from sulfate species bands between 990–1035 cm^−1^ and 1100–1180 cm^−1^,^52,53^ which points to the purity of CMCP. Moreover, when a ligand coordinates to a metal atom, new modes of vibration that are not present in the free ligand may become infrared or Raman active.^54^ Additionally, the N–H stretching frequency should shift, indicating a change of the n-electron density on nitrogen,^55^ which will be investigated thereafter.

**Fig. 2:**
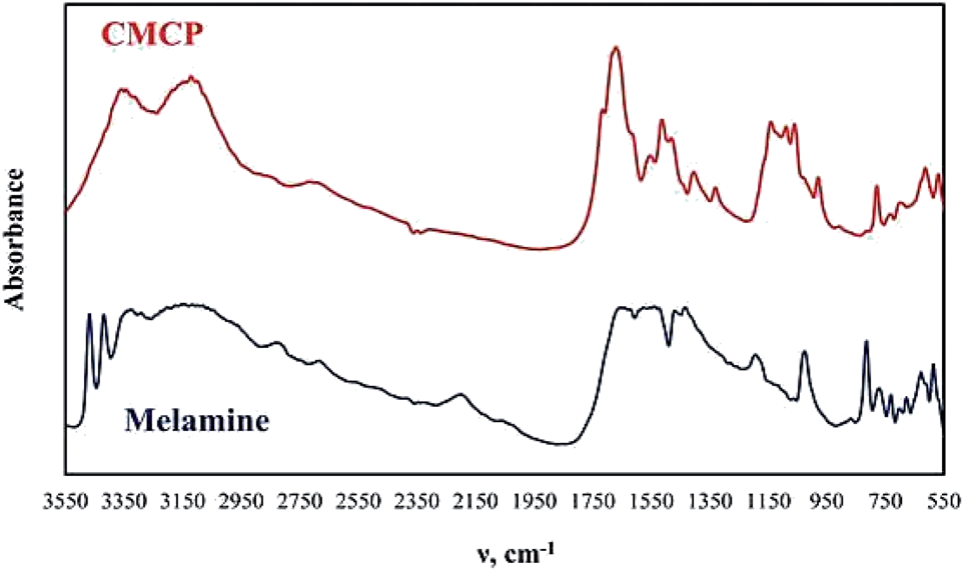
FTIR spectra of melamine and CMCP.

The FTIR analysis of melamine and CMCP is reported in Fig. 2. Melamine spectrum presents the known characteristic peaks: NH_2_ stretching (3438 and 3386 cm^−1^), asymmetric NH_2_ stretching (3243 cm^−1^), symmetric NH_2_ stretching (3111 cm^−1^), NH_2_ deformation (1614 cm^−1^), quadrant stretching of the 1,3,5-*s*-triazine ring (1515 cm^−1^), semicircle stretching of the 1,3,5-*s*-triazine ring (1425 cm^−1^), C–N stretching of primary amines (1020 cm^−1^) and out-of-plane ring bending (811 cm^−1^).^56^

On the other hand, the CMCP spectrum shows noticeable differences from that of melamine, which strongly suggests the coordination of cerium ion with melamine pendent-amines, and these are: the absence of NH_2_ stretching, the shift of both symmetric NH_2_ stretching and asymmetric NH_2_ stretching to lower wavenumbers 3333 and 3113 cm^−1^ respectively, and the shift of NH_2_ deformation to a higher wavenumber 1662 cm^−1^. At the same time, the quadrant stretching of the 1,3,5in the triazine ring are the sites that should preferably coordinate with Ce(iii),^57^ in our case, the coordination in CMCP occurred through pendent amine groups under the applied acidic condition (through optimization of acetic acid to water ratio). The proposed mechanism for this coordination presupposes that the hydrogen bonds between melamine molecules break down, and melamine molecules dissolve as melamium ions, whereby the protonation of the *N*-triazine atoms occur. As a consequence, the amino groups, conserving free lone pairs, can coordinate with Ce(iii).^57^

**Fig. 3:**
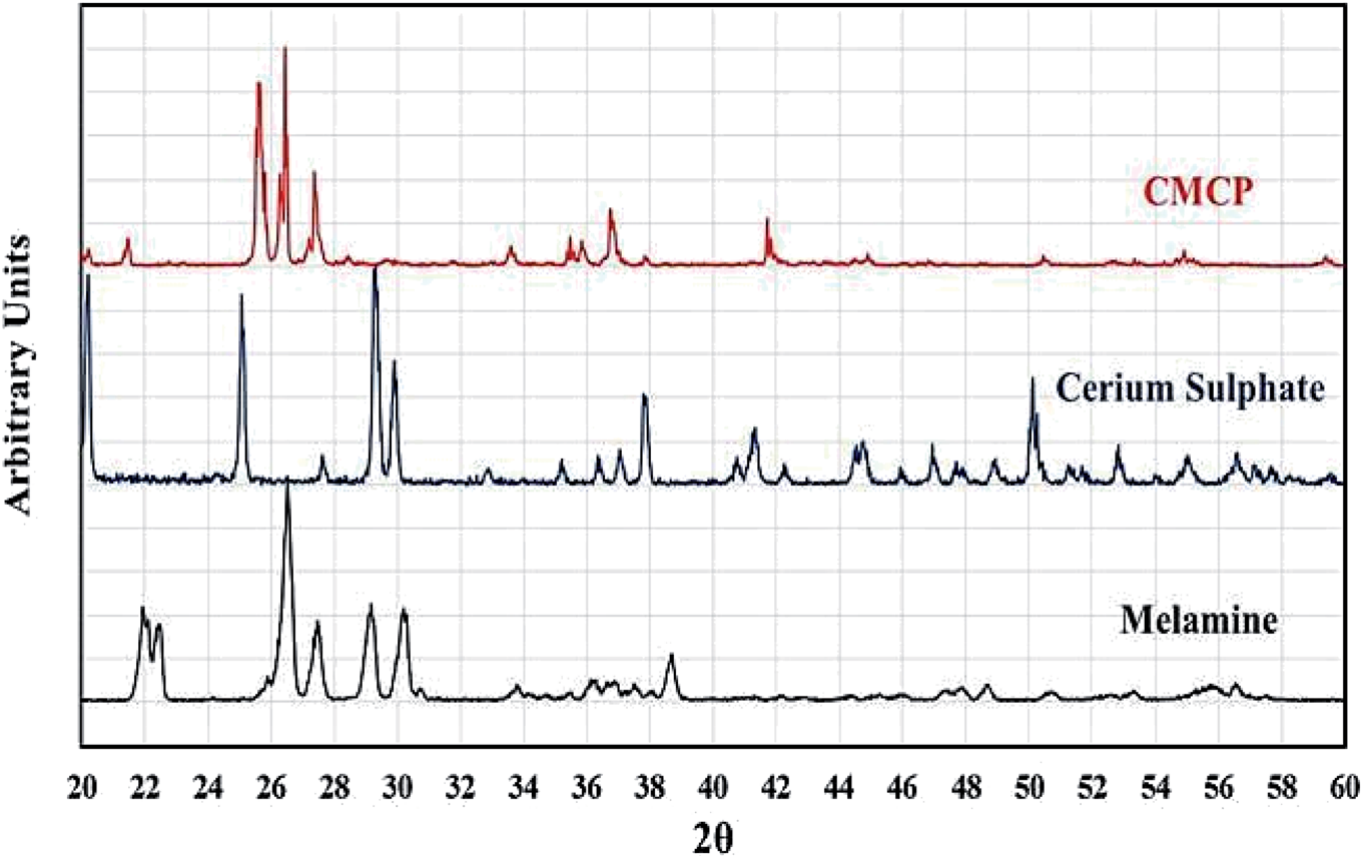
PXRD patterns of CMCP, cerium sulfate (Ce_2_(SO_4_)_3_), and melamine.

Fig. 3 shows the PXRD patterns of melamine and CMCP. For melamine, all recorded diffraction peaks are as references: 2*θ* = 22.37, 22.51, 26.89, 27.55, 29.25, 30.31, and 38.75.^58–60^ The pattern of CMCP clearly displays strong and narrow peaks, indicating the good crystallinity of the CMCP. The peaks are at 2*θ* = 7.27, 15.98, 17.53, 25.28, 26.23, and 27.23. This set is different from that of melamine and indicates the successful preparation of the crystalline CMCP. In addition, there are no peaks of CeO_2_.^61^

**Fig. 4:**
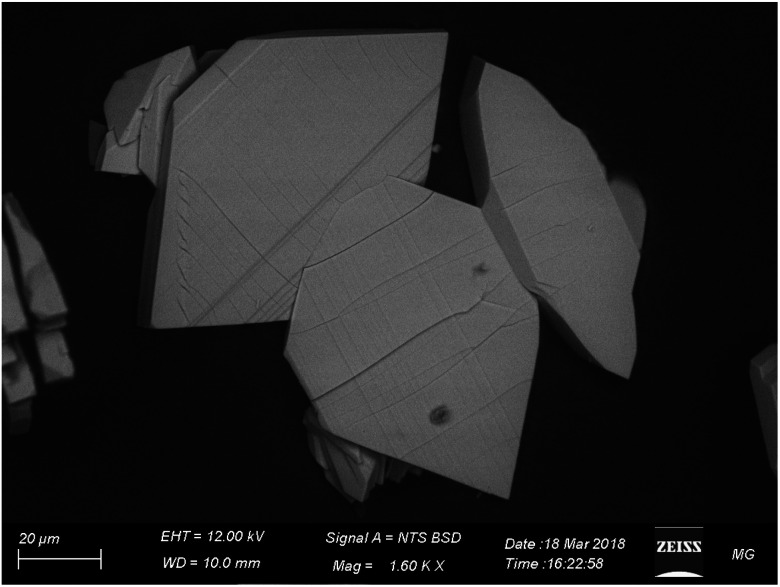
SEM image of the rhombic CMCP particles.

Fig. 4 displays the SEM image of CMCP particles. It is evident that the particles are rhombus-like thick flakes. The results of the EDX and the CHNO/S elemental analysis (Table 1) are very similar and lead to the conclusion that the Ce(iii) coordination number is eight.

**Table 1:** The relative percentage for the CMCP elemental analysis

Element	Atom% (EDX)	Atom% (EA)
H	—	22.85
C	16.29	11.42
N	32.53	22.85
O	37.85	34.28
S	9.23	8.57
Ce	4.12	—

According to the results of EDX, CHNO/S elemental analysis and FTIR, we proposed that the crystal superstructure of CMCP is of general formula [Ce^III^_2_(M)_2_(SO_4_)_3_(H_2_O)_2_·2H_2_O]_*n*_, where Ce(iii) ions are linearly coordinated to two lateral tetradentate sulfate ions forming a 1D-linear chain, while the third tetradentate sulfate ion bridges between two Ce(iii) ions from two adjacent chains forming a double-strand (or stair-like) 1D-linear chain. Each Ce(iii) ion is also coordinated to one water molecule plus one melamine molecule through the nitrogen of the amino group forming a coordination sphere of eight. Last, there are two free water molecules per each two Ce(iii) ions located in the crystal cavities. As a result, water molecules can easily diffuse between the layers and between the chains of the layers leading to dissolution of the material.^13,62^

**Fig. 5:**
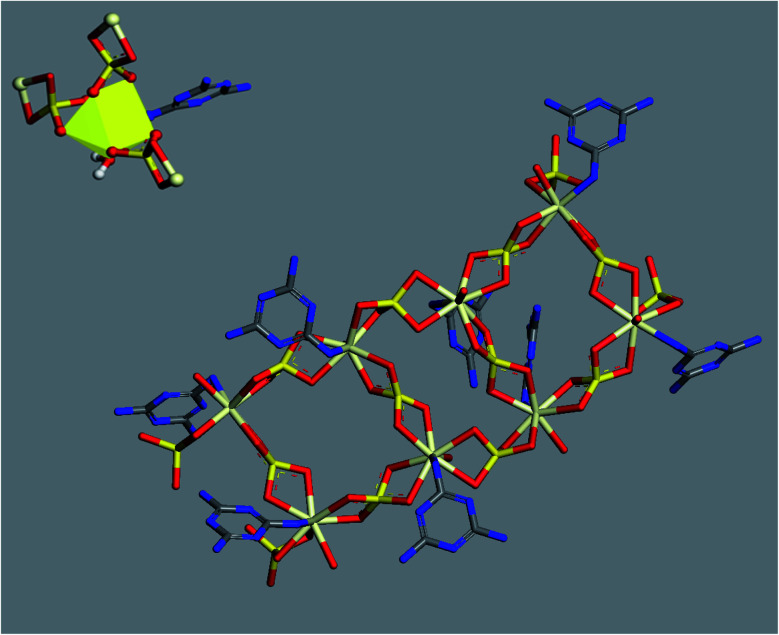
Optimized molecular structure of Ce(iii) coordination sphere (top left) and double-strand chain (centre) (hydrogen atoms were omitted for clarity).

To visualize the molecular structure of the developed material and to investigate the mechanism of assembling these 1D linear chains into the observed 3D structure, we used conformers and Forcite modules available in Biovia Materials Studio 2017 package. All simulations were done using COMPASS forcefield theory and atom-based method for summation of electrostatic and van de Waals forces. The charges were assigned using QEq method (QEq_charged 1.1) using formal charges as initial guess values and convergence limit of 1.0 × 10^−6^*e*. Geometry optimization were performed using Smart algorithm with convergence limits of 0.001 kcal mol^−1^ Å^−1^ and 1.0 × 10^−5^ Å for force and displacement respectively. The free water molecules in the structure were ignored in simulations. To construct a preliminary molecular structure for the developed material, we started by drawing the coordination sphere of Ce(iii) and running conformers module to search for the best conformer with minimum energy. Then, we continued to construct a double strand chain by connecting eight geometry optimized coordination spheres and ran geometry optimization using Forcite module to minimize the energy of the structure. The results of this simulation are displayed in Fig. 5.

**Fig. 6:**
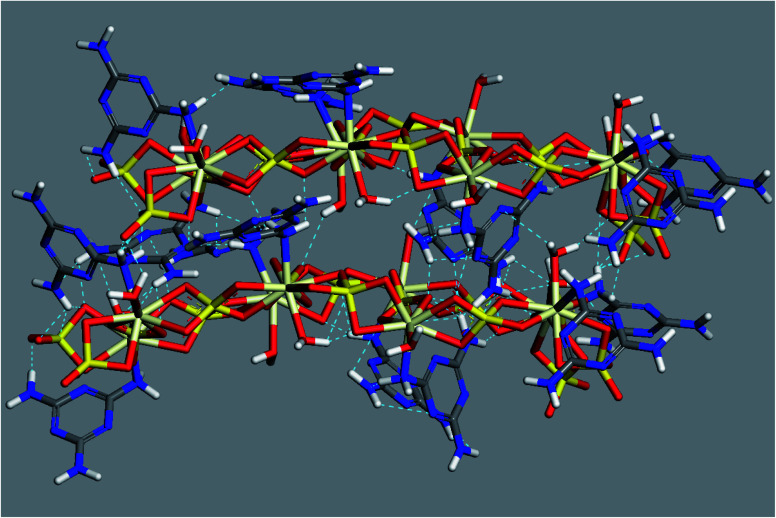
Optimized geometry and hydrogen bonding scheme of two adjacent double-strand chains (without inclusion of free water molecules).

**Fig. 7:**
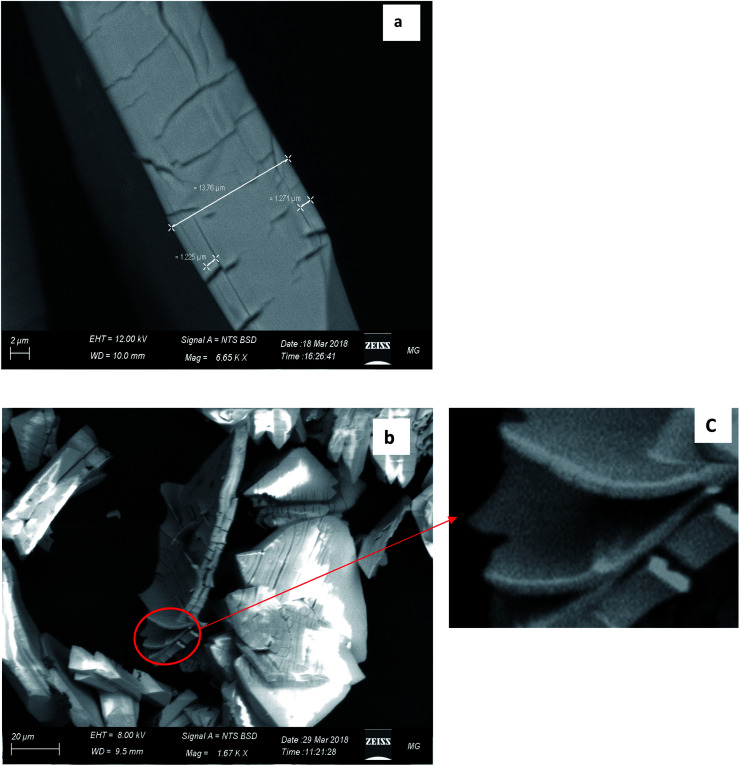
Detailed SEM images of CMCP. (a) Expressing the thickness of a single rhombus-shaped particle. (b) and (c) The exfoliation of the CMCP sheets *via* the SEM electron beam.

Further simulation was performed to investigate the way of assembling the chains into 3D structure. Two double-strand chains were inserted at close proximity (5 Å separation) and geometry optimization were carried out again to find out the best way of packing and ordering of these chains and to search for the formed hydrogen bonds as well. The image in Fig. 6 shows that the two stair-like chains are connected to each other through numerous hydrogen bonds between N(H)–O, O(H)–N, O(H)–O and N(H)–NH in the range of 2.01–3.3 Å. These hydrogen bonds in addition to the hydrogen bonds formed by the free water molecules present in the residual cavities of the structure are responsible for assembling and packing the 1D stair-like chains into 2D layers which are further assembled by the same mechanism into the observed 3D flakes. The rupture of some layers, each of which has a thickness of about 1.2 μm in Fig. 7a, after exposing the particle to an SEM electron beam for about 2 minutes, supports this 3D-topology formation mechanism. After a prolonged exposure of about 7 minutes to the SEM electron beam, two facial layers (seems to be sheets-stacked) detached from each other (Fig. 7b and c). Therefore, the electron beam energy is sufficient to heat up the sample inducing thermal stresses and/or water molecules removal resulting in rupture of some H-bonds and separation of the stacked layers.

### 3.2 Corrosion measurements of AA2024 in 3.5% NaCl

The inhibition effect of CMCP on AA2024 corrosion in 3.5% NaCl solution was investigated by potentiodynamic polarization and electrochemical impedance spectroscopy techniques as given below. As a matter of comparison, the corrosion performance of a blank, melamine, Ce(iii) sulfate (Ce), and CMCP containing solutions were also considered.

**Fig. 8:**
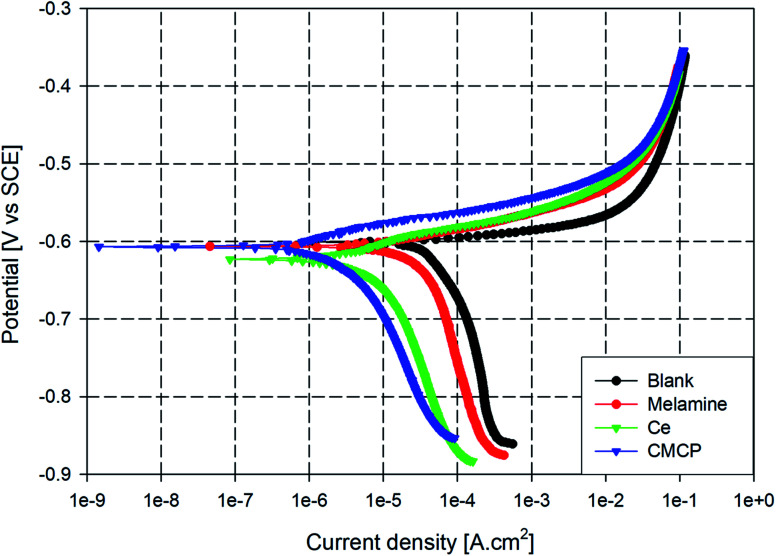
Tafel plots of AA2024 in 3.5% NaCl with different inhibitors at room temperature. Ce = Ce(iii) sulfate, CMCP = cerium(iii)–melamine coordination polymer.

The AA2024 samples were separately immersed in NaCl solutions containing 10 ppm of the different inhibitors: blank, melamine, Ce, and CMCP. The immersion time and bath temperature were 1 h and 25 °C, respectively. The polarization curves of the different samples were obtained, and the associated corrosion kinetics parameters of Tafel extrapolations (*E*_corr_, *β*_c_, *β*_a_, *I*_corr_, and IE%) were determined. Fig. 8 displays the polarization diagrams of the different samples and demonstrates the anodic and cathodic polarization behavior of AA2024.

It can be noticed that the corrosion potentials of M, Ce, and CMCP seem to be in the same range as the blank sample, however, nobler. The anodic branch is very similar for all the samples, which corresponds to the oxidation of aluminum. Also, the anodic current density immediately increases sharply in the anodic polarization direction beyond the corrosion potential in all samples, and no passive region is observed. This behavior with low anodic overpotentials indicates that these samples would show localized corrosion during an immersion course in 3.5% NaCl solution, *i.e.*, pitting.

On the other hand, all curves are consistent with a cathodic process whereby oxygen controls the reduction reaction. Also, all the four samples exhibited a similar cathodic polarization response in the given condition, which indicates that all the samples have typically the same cathodic reaction but with different rates. Moreover, adding melamine insignificantly decrease the cathodic polarization of AA2024, whereas Ce and CMCP significantly suppressed the cathodic reaction. The parallel cathodic branch of CMCP and Ce samples with the blank sample indicates that CMCP inhibited the cathodic reaction but did not alter it. These data point out that the CMCP acts as a cathodic inhibitor for AA2024 in a 3.5% NaCl solution.^63–65^

Although the used CMCP contains the same amount of cerium as Ce and the same amount of melamine as M, it exhibits higher corrosion protection. The actual role of cerium in the corrosion inhibition process mainly depends on the oxidation state of cerium ion (*i.e.*, Ce^3+^ or Ce^4+^). Literature data report that the only presence of Ce(iii) ion can provide corrosion protection.^66^ On the other hand, melamine molecule contains six nitrogen atoms, which could enact an adsorptive-blocking mechanism of the aluminum substrate.^67^ However, the excessive hydrogen bonding in melamine structure makes its dissolution difficult.

Table 2 lists the electrochemical kinetic parameters of the Tafel extrapolations (*E*_corr_, *β*_c_, *β*_a_, *I*_corr_, IE%). The anodic and cathodic slopes (*β*_a_ and *β*_c_) are in the same range (Table 2). Likewise, *E*_corr_ (the corrosion potentials) are close to each other. The clear difference lies in the corrosion current density (*I*_corr_), whereby the CMCP and Ce samples are an order of magnitude lower than other samples, and the CMCP is the lowest. Although CMCP contains a lower concentration of Ce(iii) than the Ce sample, it offers better corrosion protection.

**Table 2:** Corrosion kinetic parameters of AA2024 with varying concentrations for different corrosion inhibitors. Ce = cerium(iii) sulfate, CMCP = cerium(iii)–melamine coordination polymer

Sample	*β*_a_ [mV per decade]	*β*_c_ [mV per decade]	*E*_corr_ [mV]	*I*_corr_ × 10^5^ [A cm^−2^]	IE%	*R*_p_
Blank	19 ± 3.1	242 ± 3.5	−601 ± 5.9	5.6 ± 1.2	—	90.047
Melamine	26 ± 2.3	265 ± 4.4	−603 ± 5.1	2.9 ± 2.1	48.2	290.49
Ce	27 ± 1.1	232 ± 3.6	−619 ± 3.8	0.73 ± 1.8	86.9	933.79
CMCP	35 ± 2.2	195 ± 2.6	−605 ± 4.5	0.24 ± 1.0	95.7	2748.16

Moreover, Table 2 shows the values of polarization resistance (*R*_p_) and the corresponding protection efficiency (PE%) for all tested samples where CMCP sample shows the highest corrosion resistance.

On comparing the inhibition efficiency of CMCP to other previous work that used cerium inhibitor, it turns out that the corrosion inhibition percent of CMCP seems to be better than cerium(iii) and (iv) treatment for protection of AA2024. Rodič *et al.* used different cerium salts (chloride, nitrate, acetate and sulphate) as inhibitor for AA2024 in less aggressive corrosive solution (0.1 M NaCl).^68^ Also, the corrosion current density of CMCP is in the same order of that of cerium tartrate, cerium cinnamate treatment and cerium chloride for the same alloy (1.75 × 10^−6^ A cm^−2^) in 0.05 M NaCl solution.^69–71^ In our work, it can be noted that the corrosion current density of AA2024 inhibited with CMCP is slightly better than that of the synergistic effect of using cerium and melamine due to the intimate presence of cerium and melamine within same molecular structure.^72^

To better understand the corrosion performance of CMCP and to emphasize the importance of the coexistence of cerium and melamine in the same molecular structure, electrochemical impedance spectroscopy (EIS) measurements were performed. Again, as a matter of comparison, EIS was measured for blank, melamine, Ce, and CMCP samples.

Generally, AA2024 is susceptible to pitting corrosion in chloride-containing electrolytes due to inhomogeneity of the alloy surface. This heterogeneity comes from intermetallic particles (IMP), which are different in the corrosion potential with respect to the aluminium matrix. The pitting shape and size vary according to the size and nature of the IMP.^73^

IMP covers ≈3% of the AA2024 surface area, and it can be classified into three categories according to size: hardening precipitates, dispersoid particles, and inclusions. The later type not only (size >1 μm) contribute to the mechanical properties of the alloy, but also have an adverse impact on the corrosion protection.^74,75^ Particularly, IMP containing Cu (such as Al_2_CuMg, AlCuMnFe, and AlCu_2_) decreases the corrosion resistance of AA2024.^76,77^

The reduction reaction in this aerated neutral electrolyte is the oxygen reduction:

5 ½O_2_ + H_2_O + 2e^−^ → 2OH^−^

While Al undergoes a series of oxidation reactions in this chloride-containing solution, forming an aluminium hydroxyl chloride complex that easily dissolves in the solution.

6 Al + 3H_2_O → Al(OH)_3_ + 3H^+^ + 3e^−^

7 Al + 2H_2_O → AlO(OH) + 3H^+^ + 3e^−^

8 Al + 

<svg xmlns="http://www.w3.org/2000/svg" version="1.0" width="18.545455pt" height="16.000000pt" viewBox="0 0 18.545455 16.000000" preserveAspectRatio="xMidYMid meet"><metadata>
Created by potrace 1.16, written by Peter Selinger 2001-2019
</metadata><g transform="translate(1.000000,15.000000) scale(0.015909,-0.015909)" fill="currentColor" stroke="none"><path d="M80 840 l0 -40 -40 0 -40 0 0 -40 0 -40 40 0 40 0 0 40 0 40 120 0 120 0 0 -40 0 -40 -80 0 -80 0 0 -40 0 -40 80 0 80 0 0 -80 0 -80 -120 0 -120 0 0 40 0 40 -40 0 -40 0 0 -40 0 -40 40 0 40 0 0 -40 0 -40 120 0 120 0 0 40 0 40 40 0 40 0 0 80 0 80 -40 0 -40 0 0 40 0 40 40 0 40 0 0 40 0 40 -40 0 -40 0 0 40 0 40 -120 0 -120 0 0 -40z M720 840 l0 -40 -40 0 -40 0 0 -80 0 -80 -40 0 -40 0 0 -80 0 -80 -40 0 -40 0 0 -80 0 -80 -40 0 -40 0 0 -80 0 -80 -40 0 -40 0 0 -40 0 -40 -40 0 -40 0 0 -40 0 -40 80 0 80 0 0 40 0 40 40 0 40 0 0 80 0 80 40 0 40 0 0 80 0 80 40 0 40 0 0 -40 0 -40 80 0 80 0 0 40 0 40 40 0 40 0 0 -80 0 -80 -40 0 -40 0 0 -40 0 -40 -40 0 -40 0 0 -40 0 -40 -40 0 -40 0 0 -40 0 -40 200 0 200 0 0 40 0 40 -80 0 -80 0 0 40 0 40 40 0 40 0 0 80 0 80 40 0 40 0 0 40 0 40 -40 0 -40 0 0 40 0 40 -120 0 -120 0 0 -40 0 -40 -40 0 -40 0 0 80 0 80 40 0 40 0 0 80 0 80 40 0 40 0 0 80 0 80 -40 0 -40 0 0 -40z"/></g></svg>

H_2_O → Al_2_O_3_ + 3H^+^ + 3e^−^

9 Al(OH)^2+^ + Cl^−^ → Al(OH)Cl^+^ + 3H^+^ + 3e^−^

The presence of IMP increases the area of oxygen reduction and the corrosion process takes place in the IMP neighbouring area, forming pitting.

**Fig. 9:**
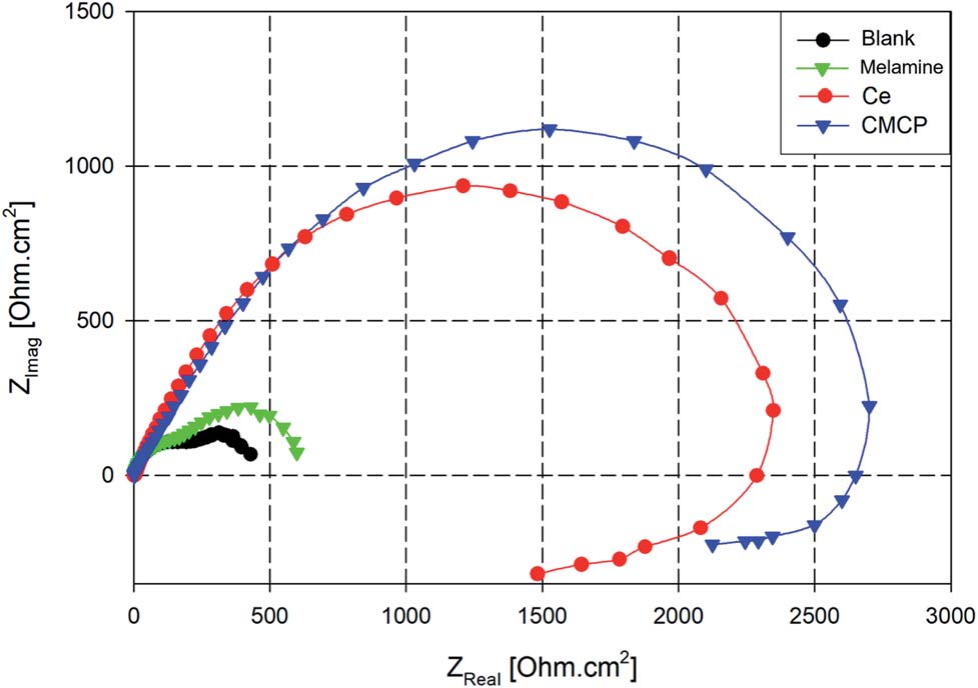
Nyquist plots AA2024 in 3.5% NaCl solution with different additives. Ce = Ce(iii) sulfate, CMCP = cerium(iii)–melamine coordination polymer.

Nyquist plots of AA2024 in a 3.5% NaCl solution after 24 h at open circuit potential, either in the absence or presence of various additives, are reported in Fig. 9. All the plots are imperfect semicircles with different diameters. This deviation from perfectness may be due to the frequency dispersion and/or the inhomogeneity of the surface.^78,79^ In a Nyquist plot, the greater the real diameter of the Nyquist' loop, the higher the corrosion resistance, where this radius represents the total resistance, including the charge transfer resistance (*R*_ct_) of the corrosion process.

In Fig. 9, the blank sample exhibits the smallest diameter in the Nyquist plot, and the melamine's diameter is slightly greater, *i.e.*, it acts slightly better as a corrosion inhibitor. In contrast, the CMCP and Ce samples have superior corrosion protection.

Also, the impedance plots of the blank and melamine have two semicircles; a capacitive loop that covers the high range of frequency and another loop at the low-frequency range. For aluminium alloys, this loop at the high-frequency range is attributed to formation of an oxide layer.^80,81^ Aluminium surface is immediately oxidized by O_2_ in air or aqueous solutions.^82^ However, the other one (time constant) at low frequency usually associates with the dissolution of aluminium *i.e.* evolution of corrosion products.^63,83^ These results are in good agreement with the potentiodynamic polarization results and the visual inspection where dark spots appeared on the surfaces of both samples after 72 h of immersion as indication of dissolution of AA2024.^84^

While the Nyquist plot CMCP and Ce samples show two impeded capacitive loops at high and mid frequency range, in addition to an inductive loop at low frequency range. The presence of an inductive loop at a low frequency may be attributed to redox activity^85,86^ and/or adsorption/desorption process of intermediates taking place on the surface of the substrate.^87,88^ The capacitive loop at high frequency range represents the inhibitive and/or the oxide film, however, the other loop represents the double-layer capacitance and the charge transfer resistance.

**Fig. 10:**
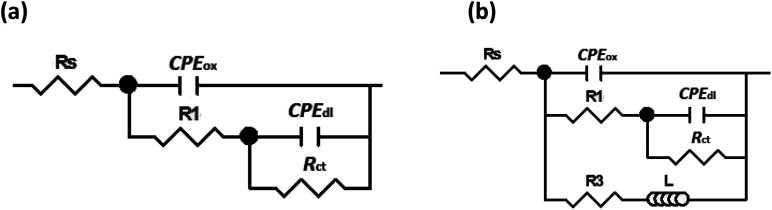
Equivalent circuits used for modelling of impedance data for (a) blank and melamine (b) CMCP and Ce samples in 3.5% NaCl solution at room temperature.

EIS is a semi-quantitative technique that offers measuring and predicting the protection efficiency of a metal in a corrosive electrolyte.^89–91^ The quantitative analysis of the EIS technique was applied by fitting the experimental data into an equivalent circuit. The equivalent circuit illustrated in Fig. 10a is used to fit the experimental data of both blank and melamine samples in the NaCl solution. While, equivalent circuit depicted in Fig. 10b is used for CMCP and Ce samples. The circuit, Fig. 10a, composes of ohmic electrolyte resistance (*R*_s_), in series with a first constant phase element for the oxide film (CPE_ox_), in parallel with a second resistance (*R*_1_) representing resistance of the oxide film, and in series with a second circuit correlated to the interaction at the metal/electrolyte interface. This interaction is represented by the double-layer capacitance (CPE_dl_) and the charge transfer resistance (*R*_ct_). A constant phase element (CPE) are used instead of pure capacitance due to defects such as surface roughness, heterogeneous appearance.^92^ In addition to the above components, circuit in Fig. 10b contains resistance (*R*_3_) and an inductor (*L*) representing the redox reaction and/or adsorbed intermediates on a fresh substrate.

The fitting results of the experimental EIS data are listed in Table 3. It is clear from the table that the charge transfer resistance values of Ce and CMCP samples are much greater than that of blank and melamine samples.

**Table 3:** Fitting results for the EIS measurements of AA2024 performed in 3.5% NaCl with different additives

Sample	*R*_s_ [Ω cm^2^]	CPE_ox_		*R*_1_ [Ω cm^2^]	CPE_dl_		*R*_ct_ [kΩ cm^2^]	*R*_3_ [Ω cm^2^]	*L* [kH cm^−2^]
		*Y* [μΩ^−1^ cm^−2^ s^*n*^]	*n*		*Y* [μΩ^−1^ cm^−2^ s^*n*^]	*n*			
Blank	5.2	1362.1	0.87	185.1	86.3	0.90	0.26	—	—
Melamine	7.4	113.4	0.89	192.3	9.9	0.93	0.42	—	—
Ce	8.1	194.5	0.91	128.6	6.4	0.94	2.11	241.3	17.6
CMCP	8.9	121.5	0.93	201.5	5.2	0.95	2.58	521.1	22.7

### 3.3 Morphology of the corrosion of AA2024 in 3.5% NaCl

**Fig. 11:**
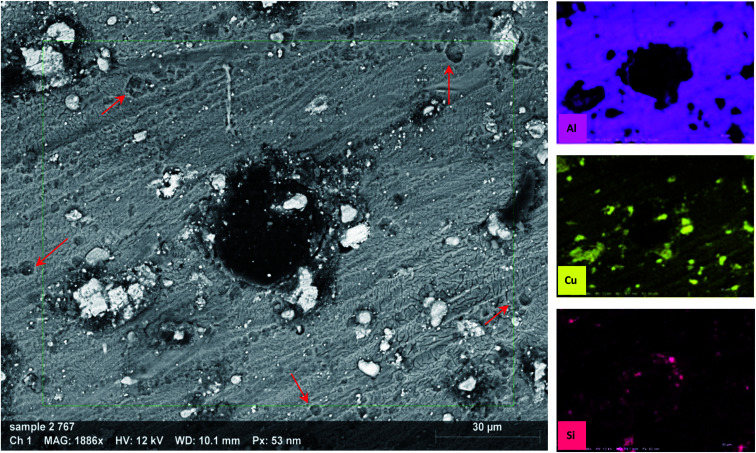
SEM images and EDX analysis of AA2024 tested in 3.5% NaCl after 72 h at room temperature.

An SEM equipped with EDX imaged the morphology of the samples after 72 h of immersion in 3.5% NaCl electrolyte (Fig. 11). The back-scatter image shows severe pitting covering the whole surface. It can be noticed from the images that there are two types of pitting on the sample surface; small (red arrow) and large (the one in the center). The former can be attributed to IMP that detached from the surface in an earlier stage of immersion, forming a honeycomb-like shape.^63,93^ The latter seems to propagate in the area adjacent to IMP containing Cu, as the EDX analysis indicates.

**Fig. 12:**
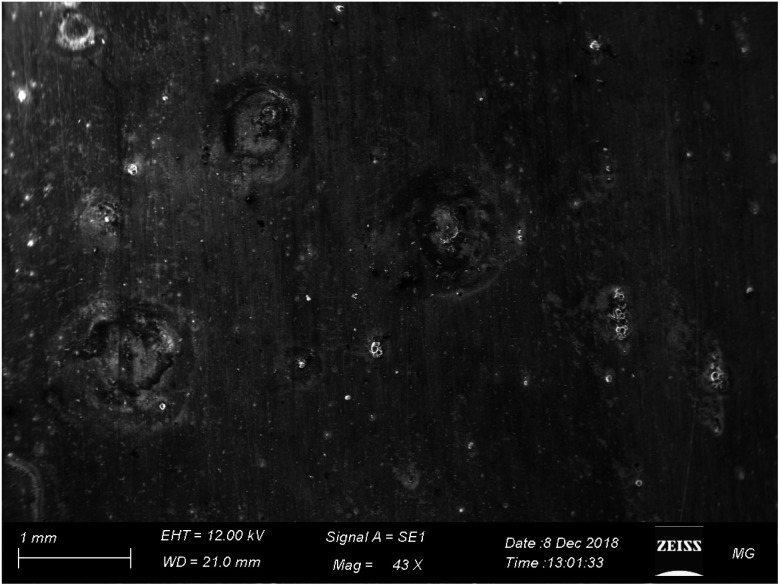
SEM images of AA2024 tested in 3.5% NaCl after 72 h melamine at room temperature.

Fig. 12 displays the SEM images of AA2024 in the 3.5% NaCl solution with 10 ppm melamine. The surface exhibits many pits of different sizes covering the whole surface. This result proves that melamine alone cannot protect the alloy from corrosion at the tested conditions, in agreement with the electrochemical measurements.

**Fig. 13:**
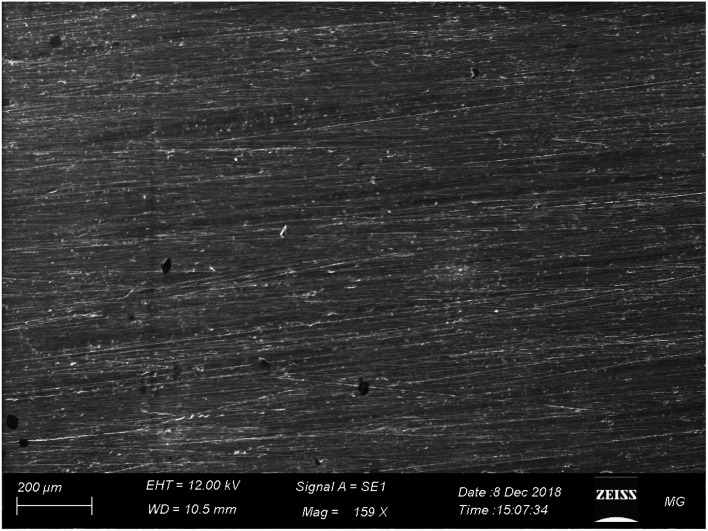
SEM images of AA2024 tested in 3.5% NaCl after 72 h with Ce(iii) at room temperature.

Ce(iii) sulfate showed better corrosion protection of AA2024 in 3.5% NaCl solution than melamine (Fig. 13), where neither pitting nor corrosion products appeared after 72 h of immersion in the corrosive solution. These findings seem to be consistent with other data, which found that Ce(iii) can protect AA2024 in 3.5% NaCl.^66,94,95^

**Fig. 14:**
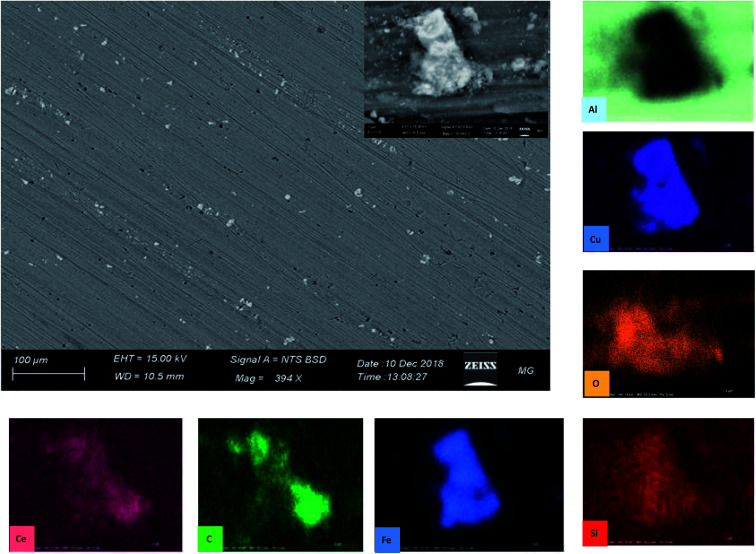
SEM images of AA2024 tested in 3.5% NaCl after 72 h with CMCP at room temperature.

Fig. 14 reports the SEM image of AA2024 after 72 h of immersion in the 3.5% NaCl solution in the presence of 10 ppm CMCP. It is clear from images, at both low and high magnification, that there is no sign of corrosion on the surface of the sample. Besides, the high-resolution image demonstrates an intermetallic particle, around which there is no sign of corrosion. The EDX mapping also shows that the IMP contains copper, silica, and iron, where it is, generally, nobler than the aluminum matrix. The EDX clearly shows that cerium, oxygen, and carbon cover the IMP, where the source of carbon, in this case, is melamine. Therefore, the EDX analysis manifests the electrochemical measurements.

In conclusion, CMCP offers corrosion inhibition for AA2024 by forming a film that covers the cathodic sites, which decreases the oxygen reduction reaction and consequently decreases the susceptibility to pitting corrosion.

### 3.4 Extended time (15 days) corrosion measurements of AA2024 in 3.5% NaCl

**Fig. 15:**
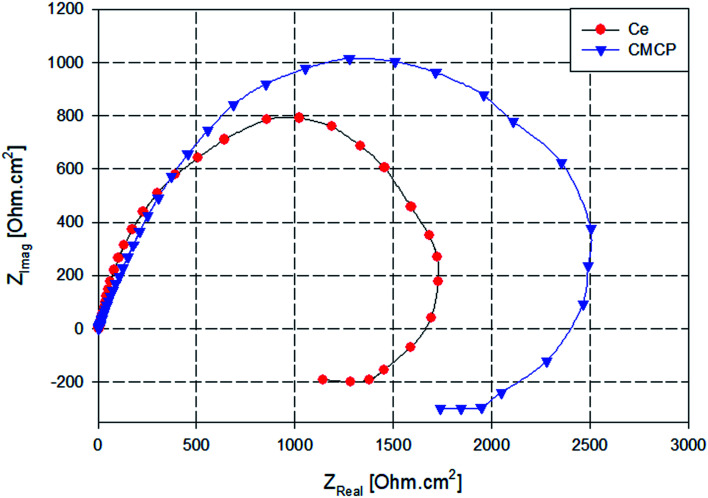
Nyquist plots of AA2024 with Ce and CMCP in 3.5% NaCl solution after 15 days of immersion.

The data above shows that both Ce and CMCP offer comparable corrosion prevention for AA2024 in artificial seawater. Nevertheless, upon an extended time of immersion, CMCP exhibits a more stable behavior than that of Ce(iii) (Fig. 15). Neither Ce nor CMCP sample change its corrosion behavior during the immersion period. However, they lose some of their impedance. The polarization resistance, *R*_p_, can be estimated as the impedance (frequency → 0) at −*Z*_Imag_ = 0.^96,97^ Indeed Ce(iii) loses nearly ≈20% of the polarization resistance, while CMCP loses nearly ≈9%. These results indicate that CMCP can offer more stable corrosion protection than that of Ce sample.

CMCP can offer corrosion prevention to the AA2024 *via* the following assumed mechanism; the aluminium matrix (anode) is oxidized following eqn (6) and (7).^98^ The Ce(iii) within the CMCP (stair-like chains) reacts with the hydroxide ions to form a thin hydroxide layer of Ce(iii) (eqn (10)). The presence of H_2_O_2_ as an intermediate of oxygen reduction (eqn (11)) would interact with Ce(iii) to precipitate Ce(iv) at the cathodic sites (the intermetallic particles) according to eqn (12).^66,98^ Generally, Ce(iii) can be oxidized to Ce(iv) at high pH as a result of the OH^−^ ions (eqn (5) and (11)). In this case, the CMCP would hinder the cathodic reaction (oxygen reduction) taking place at the cathodic sets, consequently, decreasing the corrosion rate of the aluminium alloy.^72^ This proposed mechanism is in good agreement with the PDP results, where the results indicated that the type of corrosion inhibitor is predominantly a cathodic inhibitor.

10 Ce^3+^ + 3OH^−^ → Ce(OH)_3_

11 O_2_ + H_2_O + 2e^−^ → H_2_O_2_ + OH^−^

12 2Ce^3+^ + H_2_O_2_ → 2Ce^4+^ + 2OH^−^

Melamine in the molecular structure of the CMCP offers different types of protecting actions, as follows: (i) it is adsorbed on the metal substrate *via* nitrogen atoms due to the lone-pairs to coordinate to the metal surface.^99,100^ (ii) It improves the interaction of such a large molecule (coordination polymer), as it acts as a structure-director.^101^ (iii) It reduces Ce(iv) to Ce(iii), where melamine should be oxidized subsequently.^102,103^ In the last case, melamine often regenerates Ce(iii) during the immersion, thus extending the period of corrosion protection (Fig. 15).

## 4. Conclusions

Cerium(iii)–melamine coordination polymer (CMCP) was synthesized *via* one-pot mixed-solvothermal technique. Raman, FTIR, and PXRD techniques were applied to investigate the prepared coordination of cerium(iii) with melamine. It was found that the coordination occurs through –NH_2_ groups rather than the *N*-triazine ring, which is distinctive. The morphology study clarifies that CMCP particles has a rhombic shape induced by a coordination number of eight around the cerium atom. The results were in a good agreement with the computational calculations conducted by Material studio software. The CMCP structure is composed of 1D stair-like chains assembled into 2D layers, which in turn stacked into 3D flakes mainly *via* H-bonding of the free water molecules located inside the residual voids of the structure. Importantly, water molecules can easily diffuse between the layers and between the chains of the layers leading to dissolution of the material. The electrochemical measurements confirmed that CMCP inhibits corrosion and protects the aluminium alloy AA2024 in artificial seawater (3.5% NaCl) better than any of its constituents solely. The mechanism through which inhibition comprises a synergistic action by the presence of both cerium(iii) ion and melamine side by side within the same structure, which induces a see-saw redox process of cerium (continuous back-forth: Ce(iii) to Ce(iv)). Moreover, CMCP offers more stable corrosion protection then Ce(iii) alone as melamine regenerates Ce(iii) often during immersion as confirmed by the 15 days extended immersion tests.

## Conflicts of interest

There are no conflicts to declare.

## Supplementary Material
